# What is your diagnosis?

**DOI:** 10.4274/jtgga.2017.0030

**Published:** 2017-12-15

**Authors:** İlknur Adanır, Ayşe Filiz Gökmen Karasu, Banu Dane

**Affiliations:** 1 Department of Obstetrics and Gynecology, Bezmialem Vakıf University Faculty of Medicine, İstanbul, Turkey

A 31-year-old patient presented for routine first trimester antenatal screening at 11 weeks 5 days. On ultrasonographic examination, crown-rump length: 58 mm, nuchal translucency: 1.3 mm, and nasal bone and tip were present. The lower limbs were observed as fused (Figure 1, 2). A single umbilical artery, and an intra-abdominal septated cystic mass was seen.

The patient (gravida 3, para 1, abortion 1) had no history of diabetes mellitus. There was no consanguinity. She did not use alcohol or illicit drugs, but consumed tobacco (5 cigarettes per day). She had a child with Wilms’ tumor, with no mutations of the WT1 gene. Hematologic and biochemical parameters were all within the normal range. The combined test result of the patient was normal. Chorionic villus sampling was performed, and the result reported as normal.

The patient was hospitalized at 13 weeks and 4 days for termination of the pregnancy. Misoprostol was used for medical abortion. On physical examination, the fetus had fused lower limbs with no feet like a tail, absent external genitalia, imperforate anus, and a single umbilical artery (Figure 3, 4). The upper extremities were morphologically normal. Autopsy was performed. At autopsy, the external examination revealed imperforate anus, absent external genitalia, single umbilical cord, and fusion of lower extremities. Bilateral renal agenesis was also noted at autopsy.

## ANSWER

Sirenomelia or mermaid syndrome, is a congenital deformity. The prevalence is estimated to be one in 100.000 live births (1). Sirenomelia is a fatal congenital defect with characteristic features of partial or complete fusion of the lower limbs accompanying various other anomalies such as bilateral renal agenesis or dysgenesis, an imperforated anus and absence of the rectum, absence of the sacrum and other vertebral defects, absence of both external and internal genitalia, and oligohydramnios. Oligohydramnios is usually the first sign of this syndrome in the second trimester. Early detection of this syndrome prenatally is very rare (2,3).

Although rare cases of sirenomelia may survive for a short time (4,5,6), sirenomelia is a fatal congenital defect, and pregnancy termination should be offered to the parents. Sirenomelia can be associated with diabetes mellitus (7), teratogen exposure (8), and drug abuse (5). In our case, the patient used tobacco during pregnancy. Diagnosis of sirenomelia is usually made during the second trimester of pregnancy, the alerting sign is mostly oligohydramnios. Due to oligohydramnios, evaluation of fetus is more difficult than in the first trimester. First trimester diagnosis of sirenomelia is more important because this is a fatal anomaly. Accordingly, first trimester screening of limb defects is important to catch sirenomelia. Our literature search demonstrated that only a few cases of sirenomelia have been diagnosed in the first trimester. We suggest routine screening of extremities in the first trimester.

## Figures and Tables

**Figure 1:**
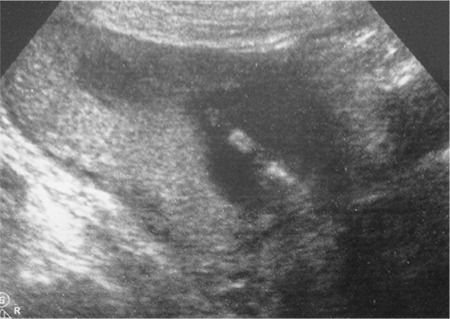
The lower limbs observed as fused

**Figure 2:**
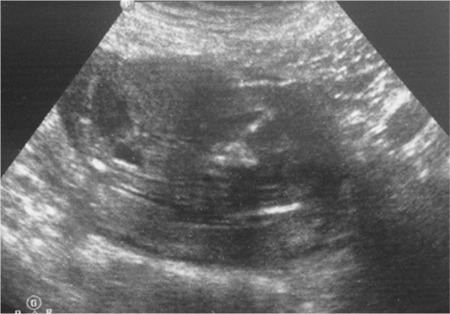
Sagittal view of the fused lower limbs

**Figure 3:**
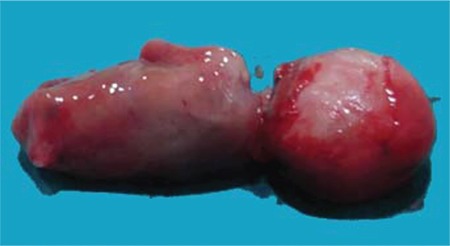
Photography of the fetus showed imperforate anus

**Figure 4:**
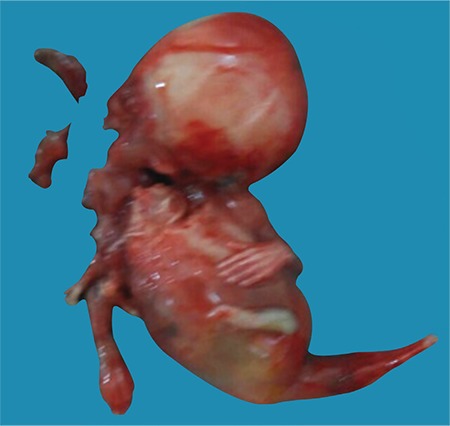
Photography of the fetus showed fused lower limbs, like a tail with no feet, absent external genitalia, and single umbilical artery
